# The impact of climatic factors on headache patterns: a 14-year time series analysis of cluster and tension-type headaches in primary care

**DOI:** 10.22514/jofph.2026.045

**Published:** 2026-07-12

**Authors:** Juan Nicolás Cuenca-Zaldívar, Carmen Corral del Villar, Silvia García Torres, Rafael Araujo Zamora, Paula Gragera Peña, Tiago José Gomes de Oliveira, Filipa Paisana Ricardo, Erika Meléndez-Oliva, Ada María González-González, Eleuterio A. Sánchez-Romero

**Affiliations:** ^1^Physical Therapy Unit, Primary Health Care Center “El Abajón”, 28231 Las Rozas de Madrid, Spain; ^2^Research Group in Nursing and Health Care, Puerta de Hierro Health Research Institute-Segovia de Arana (IDIPHISA), 28222 Majadahonda, Spain; ^3^Interdisciplinary Research Group on Musculoskeletal Disorders, 28016 Madrid, Spain; ^4^Physical Therapy Unit, Primary Health Care Center “Cerro del Aire”, 28220 Majadahonda, Spain; ^5^Physical Therapy Unit, Primary Health Care Center “San Juan de la Cruz”, 28223 Majadahonda, Spain; ^6^Sociedade Portuguesa de Disfunção Temporomandibular, Dor Orofacial e Sono, 3000-041 Coimbra, Portugal; ^7^Department of Physiotherapy, Atlantic Higher School of Health (ESSATLA), 2730-036 Barcarena, Portugal; ^8^Grupo de Investigación en Dietética Aplicada, Nutrición y Composición Corporal (DANuC), Department of Optics, Pharmacology and Anatomy, University of Alicante, 03690 Alicante, Spain; ^9^Physiotherapy and Orofacial Pain Working Group, Sociedad Española de Disfunción Craneomandibular y Dolor Orofacial (SEDCYDO), 28009 Madrid, Spain; ^10^Adavall Physiotherapy and Rehabilitation Clinic S.L, 47002 Valladolid, Spain; ^11^DENS-ia Research Group, Faculty of Health Sciences, Miguel de Cervantes European University, 47012 Valladolid, Spain; ^12^Department of Rehabilitation Sciences, Florida Gulf Coast University, Fort Myers, FL 33965, USA

**Keywords:** Headache, Cluster headache, Tension-type headache, Climatic factors, Meteoropathy, Time-series analysis, Primary care, Biometeorology

## Abstract

**Background**: Weather-related influences on pain and neurological symptoms are widely discussed in biometeorology. However, the long-term impact of climatic variables on primary care headache consultations remains unclear. This study aimed to examine whether climatic variables are associated with consultations for cluster headaches, tension-type headaches (TTH), and unspecified headaches over 14 years. **Methods**: Data (2010–2023) were extracted from medical records using International Classification of Primary Care, Second Edition (ICPC-2) codes. Meteorological variables (temperature, rainfall, wind direction, barometric pressure, and sunshine hours) were obtained from the State Meteorological Agency. Time-series analyses used Exponential Smoothing State Space Model with External Regressors (ETSX) and AutoRegressive Integrated Moving Average Models with External Regressors (ARIMAX) using age, sex, and meteorological factors as external regressors. Model accuracy was evaluated using the Root Mean Squared Error (RMSE), Symmetric Mean Absolute Percentage Error (SMAPE), and Mean Absolute Scaled Error (MASE). **Results**: A total of 5127 headache consultations were analyzed (mean age, 45.0 ± 19.9 years; 68.9% female). ETSX models best fit the overall, unspecified, and cluster headache series, whereas the ARIMAX model provided optimal performance for TTH. Sex was the strongest predictor across all models. In TTH, female sex increased consultations (*p* < 0.001), whereas higher temperature (*p* = 0.031) and wind direction (cosine component; *p* = 0.027) were associated with fewer consultations. For cluster headache, male sex was associated with fewer consultations (*p* = 0.020), and no climatic variables showed a significant association. Meteorological variables were not independently associated with unspecified headache consultations. **Conclusions**: Climatic variables showed limited, subtype-specific associations, with only temperature and wind direction independently associated with TTH. No weather variables predicted cluster headache or unspecified consultations. Demographic factors appeared to be more strongly associated with healthcare utilization than climatic variables, although ETSX and ARIMAX models may support forecasting and resource planning.

## 1. Introduction

Humans interact continuously with their environment, and climatic variability is increasingly being recognized as an important factor influencing health and disease patterns [1]. Biometeorology is the study of the impact of weather and climate on living organisms [2]. Within this field, meteoropathy refers to the manifestation of symptoms triggered or aggravated by changes in climatic conditions [3]. Headache disorders, particularly migraine and tension-type headache, represent a major global public health burden, affecting nearly three billion individuals worldwide and ranking among the leading causes of years lived with disability [4]. Furthermore, up to three-quarters of patients with chronic pain report symptom fluctuations associated with meteorological factors [5].

Barometric pressure, humidity, wind, precipitation, temperature, and solar radiation are among the most commonly studied meteorological variables associated with pain and neurological symptoms [3, 6]. These factors can alter neuroendocrine and autonomic regulation [7], influencing both mood and pain perception. In experimental models, abrupt weather changes have been associated with neuronal activation in the brainstem nuclei involved in sensory integration [7], suggesting potential mechanisms for climate-related modulation of pain.

Approximately 2.81 billion people suffered from headaches in 2021, with women aged 15–49 years being the most affected, accounting for the third leading cause of disability-adjusted life-years (DALYs) in 2021 [8]. According to the third edition of the International Classification of Headache Disorders (ICHD-3), headaches can be classified as primary or secondary [9]. Primary headaches, particularly tension-type headaches (TTHs) and cluster headaches (CHs), are among the most commonly reported meteorosensitive conditions (unspecified headaches, 14.2; tension-type headaches, 2; cluster headaches, 3.1) [9, 10]. Climatic triggers such as sudden drops in barometric pressure, temperature fluctuations, and variations in wind speed or humidity are frequently described by patients as precipitating factors [11, 12, 13]. Epidemiological studies have shown that changes in atmospheric pressure may influence the onset or intensity of migraine and tension-type headache (TTH) episodes [14, 15], whereas variations in environmental exposures and circadian factors have been associated with the temporal pattern and periodicity of cluster headache (CH) attacks [16, 17]. However, most available studies have relied on self-reported data or short observation periods, and few have employed objective long-term primary care data to systematically investigate these associations [18].

Recent large-scale analyses using smartphone applications or national registries have provided new insights into the weather–headache relationship. For example, low barometric pressure, increased humidity, and higher wind speeds have been linked to a higher frequency of headache attacks [19, 20]. A nationwide Korean study also reported that temperature variability was significantly associated with the onset of CH periods [21]. These findings highlight the potential contribution of meteorological factors to headache chronobiology and healthcare demands. Nevertheless, the heterogeneity of results across studies suggests that the magnitude and direction of these associations may differ according to population, geographic region, and headache subtype [22, 23, 24].

Despite increasing evidence, no previous study has analyzed the influence of climatic variables on weekly headache consultations in primary care using robust time-series models with external regressors over an extended period. Understanding how environmental changes affect headache-related healthcare utilization may improve clinical counseling, resource allocation, and the development of climate-sensitive predictive models.

Therefore, this study aimed to examine the association between climatic variables (temperature, precipitation, wind characteristics, hours of sunlight, and barometric pressure) and the number of primary care consultations for cluster headache and TTHs from 2010 to 2023. The secondary objectives were to explore the effects of age, sex, and headache subtype on these associations, and to analyze temporal trends in consultation rates across the 14 years.

## 2. Methods

### 2.1 Data source and study population

A retrospective cohort study was conducted using data extracted from the electronic health records of patients from three primary care centers: “El Abajón” in Las Rozas, “Cerro del Aire” in Majadahonda, and “San Juan de la Cruz” in Pozuelo de Alarcón, Spain. The study period was from 01 January 2010 to 31 December 2023, and included all patients aged 18 years and older who consulted for headache-related conditions during the study period. Diagnoses were identified using the International Classification of Primary Care, Second Edition (ICPC-2), using the following codes: N89 (unspecified headache); N01 (tension-type headache); and N03 (cluster headache). No additional subcodes (N02/N04) existed in ICPC-2; therefore, only the confirmed codes recorded in the clinical system were used.

Sociodemographic variables, including age and sex, were retrieved from electronic medical records along with the consultation date. Clinical variables included headache subtypes.

Meteorological data were obtained from the Spanish Meteorological Agency (AEMET) weather station at Pozuelo de Alarcón. The AEMET station in Pozuelo de Alarcón is located approximately 15 km from Las Rozas de Madrid and 12 km from Majadahonda, Spain. The three health areas included in this study cover a total area of 140 km^2^ (ID3194Y, latitude 40°26′54′′N, longitude 3°48′48′′W).

The study protocol was approved by the Research Ethics Committee of the Puerta de Hierro Majadahonda Hospital (PI 70/24, Act 06/2024). All procedures complied with the principles of the Declaration of Helsinki and ensured patient anonymity and confidentiality throughout the study. The study was conducted in accordance with the Strengthening the Reporting of Observational Studies in Epidemiology (STROBE) Statement and checklist [24].

All patients were included if they:

(1) were aged ≥18 years;

(2) had at least one primary care consultation registered between 01 January 2010 and 31 December 2023; and

(3) had a diagnosis coded with ICPC-2 N01 (tension-type headache), N03 (cluster headache), or N89 (unspecified headache).

Patients were excluded if:

(1) their medical record lacked age or sex information;

(2) the consultation corresponded to duplicate entries; or

(3) the diagnostic code did not belong to the headache-related ICPC-2 categories defined for this study.

### 2.2 Climatic variables

Meteorological data corresponding to the same geographical region and study period were obtained from the Spanish State Meteorological Agency (AEMET). The variables collected included mean temperature (°C), diurnal temperature range (°C), day-to-day temperature change (°C), wind direction (°), mean wind speed and wind gust speed (m/s), daily barometric pressure range and day-to-day barometric pressure change (hPa), and sunshine hours (h/day). Because wind direction is a circular variable (0° and 360° representing the same direction), it cannot be directly modeled as a linear predictor. Therefore, the wind direction was transformed into sine and cosine components, which allowed circular data to be appropriately represented in the regression models.

Daily meteorological data were aggregated into weekly values to match the frequency of medical consultations. These meteorological indicators were chosen based on prior studies suggesting that atmospheric changes, particularly temperature, barometric pressure, and humidity, may influence neurological symptoms and pain modulation [13, 19, 20, 25].

### 2.3 Follow-up process for consultations

To capture longitudinal patterns in headache-related healthcare utilization, the number of primary care consultations associated with headache diagnoses was aggregated on a weekly basis; however, individual patient trajectories were not analyzed. This longitudinal follow-up enabled the identification of repeated medical visits for each headache subtype (CH, TTH, and unspecified headache), thereby allowing the analysis of temporal fluctuations and recurrence patterns in primary care.

### 2.4 Sample size

According to Burmeister *et al*. [26], at least 240 observations are required to ensure sufficient power for a multiple regression model with 11 continuous predictors and one dichotomous predictor. The present study substantially exceeded this requirement, including 5127 consultations recorded between 2010 and 2023. Weekly data aggregation produced a time series of approximately 728 observations, allowing robust estimation of both the ETSX and ARIMAX models with external regressors. This calculation is provided for orientation only, as traditional power calculations do not directly apply to time-series models such as ETSX or ARIMAX.

### 2.5 Study procedures

Patient data, including age, sex, and ICPC-2 diagnostic codes, were anonymized and extracted from electronic medical records. These clinical variables were systematically linked to the corresponding meteorological parameters obtained from the Spanish State Meteorological Agency (AEMET) for the same observation period. The climatic dataset included mean temperature (°C), diurnal temperature range (°C), day-to-day temperature change (°C), wind direction (°) transformed into cosine and sine wind directions, mean and gust speed (m/s), daily barometric pressure range and its day-to-day change (hPa), and sunshine hours (h/day) [27].

Each variable was aggregated weekly to align with the frequency of medical consultations and ensure consistency between environmental exposure and health care activities.

### 2.6 Outcome measures

The primary outcome was the weekly number of primary care consultations for headaches, subdivided into tension-type, cluster, and unspecified headaches, as recorded using ICPC-2 diagnostic codes.

The secondary outcomes included the evaluation of temporal trends, sex- and age-related differences, and the influence of climatic variables on consultation frequency. Diagnostic criteria were consistently applied across 14-year period, and meteorological variables were defined according to the AEMET operational standards to ensure homogeneity and reproducibility across all data sources.

### 2.7 Statistical analysis

Statistical analysis was performed using R version 4.1.3 (R Foundation for Statistical Computing, Institute for Statistics and Mathematics, Welthandelsplatz 1, 1020 Vienna, Austria).

The significance level was set at *p* < 0.05.

Quantitative variables are described as mean ± standard deviation, and qualitative variables are presented as absolute and relative frequencies (%).

The number of cases was analyzed from 01 January 2010 to 31 December 2023 using a time-series analysis, with weekly counts of CH, unspecified headache, and TTH. External regressors included age, sex, mean temperature, diurnal temperature range, day-to-day temperature change, wind direction, mean wind speed, and gust speed, as well as daily barometric pressure range and its day-to-day change, after excluding variables with a variance inflation factor (VIF) greater than 10. In the case of wind direction, because it is a circular variable where North is both at 0° and 360°, it was segmented into the cosine and sine of the wind direction and represented using sine and cosine components to appropriately model circular data [28]. The model selection, Exponential Smoothing State Space Model with external regressors (ETSX), or AutoRegressive Integrated Moving Average with external regressors (ARIMAX), was carried out by analyzing the accuracy of the predictions of a training set (75% of the sample) on a test set (25% of the sample) and comparing the root mean squared error (RMSE), the Symmetric Mean absolute percentage error (SMAPE), and the mean absolute scaled error (MASE), in which the lower the value, the better, between both [29, 30] selecting the model with the best overall value across the three statistics. The stationarity of the series was tested using the Augmented Dickey-Fuller test, as well as compliance with the assumptions using the Ljung-Box test (autocorrelation of the residuals) and the Kolmogorov-Smirnov test with Lilliefors correction (normality of the residuals).

Due to the presence of 14 weeks in which there were missing data in the variables age, sex (male and female), CH, Headache, and TTH, which represents 1.92% of the series, missing data were imputed using the Predictive Mean Matching (PMM) method implemented in the mice package in R [31] to ensure analytical consistency. All statistical procedures were double-checked for reproducibility and internal consistency.

## 3. Results

A total of 5127 headache-related consultations were included in the study, comprising 1162 TTH cases (22.7%), 99 CH cases (1.9%), and 3792 unspecified headache cases (73.9%). The mean age of patients was 45.0 ± 19.9 years, and the majority were female (68.9%) (Table 1).

**Table 1.** Sample characteristics and atmospheric conditions.

	Overall	2010	2011	2012	2013	2014	2015	2016	2017	2018	2019	2020	2021	2022	2023
Socio-demographic characteristics															
Age	45.00 ± 19.91	50.31 ± 17.59	51.71 ± 18.16	50.85 ± 17.69	51.04 ± 17.26	46.57 ± 16.49	48.38 ± 17.84	46.58 ± 19.30	46.20 ± 20.46	45.84 ± 18.57	45.63 ± 18.97	42.27 ± 20.86	41.90 ± 20.03	41.47 ± 20.45	44.23 ± 21.28
Sex (Female), n (%)	3535 (68.95)	66 (54.10)	90 (62.50)	104 (63.03)	140 (73.68)	146 (67.59)	172 (72.27)	242 (74.69)	214 (73.29)	245 (68.25)	276 (74.19)	292 (68.54)	398 (67.00)	494 (68.61)	656 (67.98)
Sex (Male), n (%)	1592 (31.05)	56 (45.90)	54 (37.50)	61 (36.97)	50 (26.32)	70 (32.41)	66 (27.73)	82 (25.31)	78 (26.71)	114 (31.75)	96 (25.81)	134 (31.46)	196 (33.00)	226 (31.39)	309 (32.02)
Headache type															
Overall cases	5127	122	144	165	190	216	238	324	292	359	372	426	594	720	965
Cluster headache, n (%)	99 (1.93)	10 (8.20)	6 (4.17)	9 (5.45)	6 (3.16)	2 (0.93)	10 (4.20)	4 (1.23)	8 (2.74)	0 (0.00)	10 (2.69)	2 (0.47)	8 (1.35)	12 (1.67)	12 (1.24)
Unspecified headache, n (%)	3792 (73.96)	48 (39.34)	66 (45.83)	70 (42.42)	108 (56.84)	116 (53.70)	110 (46.22)	174 (53.70)	174 (59.59)	241 (67.13)	284 (76.34)	362 (84.98)	530 (89.23)	630 (87.50)	879 (91.09)
Tension-type headache, n (%)	1162 (22.66)	64 (52.46)	72 (50.00)	86 (52.12)	76 (40.00)	96 (44.44)	116 (48.74)	144 (44.44)	106 (36.30)	114 (31.75)	72 (19.35)	50 (11.74)	42 (7.07)	68 (9.44)	56 (5.80)
Atmospheric conditions															
Average temperature (degrees Celsius)	14.99 ± 7.66	14.74 ± 8.41	15.53 ± 7.36	14.13 ± 7.91	13.77 ± 7.73	14.91 ± 6.74	15.28 ± 7.74	14.73 ± 7.61	15.28 ± 7.87	14.57 ± 7.80	15.12 ± 7.38	15.07 ± 7.37	14.95 ± 7.29	16.14 ± 7.93	15.69 ± 7.77
Average rainfall (L/m^2^)	1.43 ± 4.84	2.02 ± 5.44	1.26 ± 3.66	1.09 ± 4.40	1.41 ± 4.48	1.48 ± 4.46	0.92 ± 3.21	1.84 ± 4.97	0.85 ± 3.23	1.94 ± 5.46	1.26 ± 4.91	1.40 ± 4.39	1.28 ± 4.74	1.48 ± 4.22	1.79 ± 8.18
Average wind speed (m/s)	2.87 ± 1.77	2.71 ± 1.77	2.54 ± 1.61	2.91 ± 1.84	2.90 ± 1.90	3.09 ± 1.88	2.68 ± 1.72	2.73 ± 1.92	2.55 ± 1.75	3.01 ± 1.71	3.29 ± 1.91	2.96 ± 1.75	2.94 ± 1.60	2.83 ± 1.54	3.01 ± 1.73
Wind gusts (m/s)	10.03 ± 3.64	10.09 ± 3.55	9.67 ± 3.32	10.26 ± 3.60	10.52 ± 3.57	10.60 ± 3.59	9.72 ± 3.74	9.89 ± 3.67	9.94 ± 3.67	10.10 ± 3.49	10.58 ± 4.07	9.75 ± 3.79	9.90 ± 3.47	9.92 ± 3.46	9.56 ± 3.82
Sunshine hours	8.20 ± 3.98	7.79 ± 4.26	8.30 ± 4.05	8.47 ± 3.92	8.16 ± 4.02	8.10 ± 3.97	8.31 ± 3.77	7.97 ± 4.17	8.75 ± 3.59	8.05 ± 3.95	8.82 ± 3.78	7.89 ± 4.20	7.86 ± 3.93	7.93 ± 4.26	8.46 ± 3.57
Diurnal temperature range (degrees Celsius)	13.74 ± 4.93	10.94 ± 3.82	12.90 ± 4.51	14.62 ± 5.10	13.68 ± 4.85	13.28 ± 4.80	13.99 ± 4.79	13.79 ± 5.25	15.47 ± 4.60	13.29 ± 4.73	14.47 ± 5.15	13.38 ± 5.08	13.95 ± 4.92	13.90 ± 4.93	14.64 ± 4.97
Day-to-day temperature change (degrees Celsius)	0.00 ± 1.97	0.01 ± 2.05	−0.01 ± 1.77	−0.01 ± 2.03	0.01 ± 2.04	0.01 ± 1.81	0.00 ± 2.08	−0.02 ± 1.85	0.02 ± 2.15	0.00 ± 1.98	0.00 ± 2.05	−0.01 ± 2.01	0.02 ± 1.99	0.00 ± 1.91	−0.01 ± 1.87
Daily barometric pressure range (hPa)	4.38 ± 2.30	4.95 ± 2.85	4.11 ± 1.91	4.21 ± 1.81	4.60 ± 2.65	4.43 ± 2.76	4.36 ± 2.22	4.52 ± 2.29	4.44 ± 2.46	4.56 ± 2.48	4.57 ± 2.45	4.27 ± 2.11	4.11 ± 1.98	4.15 ± 1.72	4.08 ± 2.04
Day-to-day barometric pressure change (hPa)	0.00 ± 2.71	−0.01 ± 3.34	0.01 ± 2.39	0.00 ± 2.24	0.01 ± 3.03	0.00 ± 2.95	0.01 ± 2.67	−0.01 ± 2.81	0.02 ± 2.79	−0.02 ± 2.91	0.00 ± 2.89	0.00 ± 2.62	0.00 ± 2.36	0.01 ± 2.26	0.00 ± 2.46
Wind direction (cosine)	0.89 ± 0.22	0.93 ± 0.05	0.94 ± 0.05	0.94 ± 0.05	0.94 ± 0.05	0.94 ± 0.04	0.94 ± 0.05	0.94 ± 0.05	0.92 ± 0.14	0.89 ± 0.23	0.86 ± 0.27	0.83 ± 0.33	0.78 ± 0.38	0.82 ± 0.35	0.81 ± 0.33
Wind direction (sine)	0.34 ± 0.20	0.32 ± 0.15	0.32 ± 0.14	0.32 ± 0.15	0.31 ± 0.16	0.32 ± 0.14	0.31 ± 0.15	0.31 ± 0.15	0.31 ± 0.17	0.35 ± 0.20	0.37 ± 0.21	0.37 ± 0.25	0.41 ± 0.27	0.38 ± 0.26	0.42 ± 0.24

*Descriptive statistics of the sociodemographic and meteorological variables were recorded from January 2010 to December 2023. Data are presented as the mean ± standard deviation for continuous variables and as absolute and relative values (%) for categorical variables. L/m^2^, liters per square meter; m/s, meters per second; hPa, hectopascals.*

Throughout the 14-year observation period, all headache types showed a generally stable trend until 2020, followed by a marked post-pandemic increase, particularly in unspecified headache cases, which largely explains the overall increase in consultation rates (Fig. 1).

**Fig. 1.** Reported time-series cases. Weekly number of primary care consultations for unspecified headaches, tension-type headache, and cluster headache between 2010 and 2023. A stable trend was observed until 2020, followed by a post-pandemic increase, primarily driven by unspecified headache cases.

After assessing multicollinearity, all 14 explanatory variables were retained in the models (VIF <10).

Model selection identified the ETSX model as the best fit for the overall, unspecified headache, and CH series, whereas the ARIMAX model was the most accurate for TTHs (**Supplementary Table 1**).

In the ETSX models, multiplicative ETSX formulations were not suitable because of the presence of zero values, which preclude logarithmic transformation. Therefore, additive specifications were preferred, including the Additive error, No trend, No seasonality (ANN) and Additive error, Additive trend, No seasonality (AAN) models. The final model selection included the *AAN* specification for the overall and unspecified headache series and the *ANN* specification for the CH series (**Supplementary Table 2**).

The Augmented Dickey-Fuller test (*p* = 0.01) indicated that the series was stationarity. Residual diagnostics showed no autocorrelation (Ljung-Box test, *p* > 0.05), but non-normality distributions in some models (Kolmogorov-Smirnov test, *p* < 0.05) (**Supplementary Table 3**). Although some models showed non-normal residuals, their predictive performance remained robust based on the RMSE, SMAPE, and MASE values (**Supplementary Tables 1,2,3**).

In the TTH model, female sex was significantly associated with higher consultation frequency (*p* < 0.001), whereas higher average temperature was associated with lower consultation frequency (*p* = 0.031).

In the overall and unspecified headache models, both sex variables were statistically significant (*p* < 0.001), and no independent climatic predictors were identified (Table 2). In the CH model, male sex was significantly associated with lower consultation frequency (*p* = 0.020), whereas no climatic variables reached statistical significance (Table 2).

**Table 2.** Summary of final time-series models.

		Coefficient (SE)	95% CI	^a^*p* value
ARIMAX models				
Tension-type headache (AR0, I0, MA3) (SAR0, SI0, SMA0), LAG1, m52				
	Age	0.009 (SE = 0.006)	−0.004, 0.021	*Z* = 1.360, *p* = 0.174
	Sex (Female)	0.103 (SE = 0.012)	0.079, 0.128	*Z* = 8.291, ***p* < 0.001**
	Sex (Male)	0.040 (SE = 0.025)	−0.009, 0.088	*Z* = 1.604, *p* = 0.109
	Average temperature (degrees Celsius)	−0.036 (SE = 0.017)	−0.070, −0.003	*Z* = −2.161, ***p* = 0.031**
	Diurnal temperature range (degrees Celsius)	−0.042 (SE = 0.041)	−0.123, 0.039	*Z* = −1.027, *p* = 0.304
	Day-to-day temperature change (degrees Celsius)	0.006 (SE = 0.115)	−0.22, 0.232	*Z* = 0.052, *p* = 0.958
	Average rainfall (L/m^2^)	0.015 (SE = 0.034)	−0.053, 0.082	*Z* = 0.429, *p* = 0.668
	Average wind speed (m/s)	−0.191 (SE = 0.151)	−0.486, 0.105	*Z* = −1.263, *p* = 0.207
	Wind gusts (m/s)	0.108 (SE = 0.074)	−0.038, 0.253	*Z* = 1.449, *p* = 0.147
	Sunshine hours	0.095 (SE = 0.060)	−0.022, 0.212	*Z* = 1.590, *p* = 0.112
	Daily barometric pressure range (hPa)	−0.068 (SE = 0.070)	−0.205, 0.070	*Z* = −0.960, *p* = 0.337
	Day-to-day barometric pressure change (hPa)	−0.079 (SE = 0.142)	−0.358, 0.200	*Z* = −0.555, *p* = 0.579
	Wind direction (cosine)	2.724 (SE = 1.228)	0.317, 5.132	*Z* = 2.218, ***p* = 0.027**
	Wind direction (sine)	0.887 (SE = 1.202)	−1.469, 3.242	*Z* = 0.738, *p* = 0.461
ETSX models				
Overall (AAN)				
	Age	0.011 (SE = 0.034)	−0.055, 0.078	*t*(712) = 0.336, *p* = 0.737
	Sex (Female)	−0.630 (SE = 0.022)	−0.673, −0.587	*t*(712) = −28.501, ***p* < 0.001**
	Sex(Male)	−0.795 (SE = 0.094)	−0.979, −0.611	*t*(712) = −8.496, ***p* < 0.001**
	Average temperature (degrees Celsius)	0.017 (SE = 0.081)	−0.141, 0.176	*t*(712) = 0.213, *p* = 0.831
	Diurnal temperature range (degrees Celsius)	0.036 (SE = 0.217)	−0.390, 0.462	*t*(712) = 0.165, *p* = 0.869
	Day-to-day temperature change (degrees Celsius)	−0.120 (SE = 0.619)	−1.334, 1.095	*t*(712) = −0.194, *p* = 0.847
	Average rainfall (L/m^2^)	0.027 (SE = 0.180)	−0.327, 0.381	*t*(712) = 0.150, *p* = 0.881
	Average wind speed (m/s)	−0.018 (SE = 0.801)	−1.592, 1.554	*t*(712) = −0.023, *p* = 0.982
	Wind gusts (m/s)	0.011 (SE = 0.388)	−0.751, 0.772	*t*(712) = 0.028, *p* = 0.978
	Sunshine hours	−0.012 (SE = 0.307)	−0.615, 0.590	*t*(712) = −0.040, *p* = 0.968
	Daily barometric pressure range (hPa)	0.036 (SE = 0.361)	−0.672, 0.743	*t*(712) = 0.099, *p* = 0.921
	Day-to-day barometric pressure change (hPa)	0.266 (SE = 0.769)	−1.243, 1.775	*t*(712) = 0.346, *p* = 0.729
	Wind direction (cosine)	2.358 (SE = 6.561)	−10.522, 15.238	*t*(712) = 0.359, *p* = 0.719
	Wind direction (sine)	2.020 (SE = 6.370)	−10.486, 14.525	*t*(712) = 0.317, *p* = 0.751
Cluster headache (ANN)				
	Age	−0.003 (SE = 0.002)	−0.007, 0.001	*t*(714) = −1.359, *p* = 0.174
	Gender (Female)	−0.005 (SE = 0.004)	−0.013, 0.002	*t*(714) = −1.383, *p* = 0.167
	Gender (Male)	−0.018 (SE = 0.008)	−0.034, −0.003	*t*(714) = −2.326, ***p* = 0.020**
	Average temperature (degrees Celsius)	−0.003 (SE = 0.005)	−0.012, 0.007	*t*(714) = −0.543, *p* = 0.587
	Diurnal temperature range (degrees Celsius)	0.007 (SE = 0.012)	−0.017, 0.030	*t*(714) = 0.561, *p* = 0.575
	Day-to-day temperature change (degrees Celsius)	0.025 (SE = 0.038)	−0.049, 0.099	*t*(714) = 0.662, *p* = 0.508
	Average rainfall (L/m^2^)	0.001 (SE = 0.011)	−0.021, 0.022	*t*(714) = 0.078, *p* = 0.938
	Average wind speed (m/s)	−0.021 (SE = 0.047)	−0.113, 0.071	*t*(714) = −0.440, *p* = 0.660
	Wind gusts (m/s)	0.007 (SE = 0.023)	−0.039, 0.052	*t*(714) = 0.290, *p* = 0.772
	Sunshine hours	−0.007 (SE = 0.018)	−0.042, 0.028	*t*(714) = −0.419, *p* = 0.675
	Daily barometric pressure range (hPa)	−0.007 (SE = 0.022)	−0.049, 0.036	*t*(714) = −0.300, *p* = 0.764
	Day-to-day barometric pressure change (hPa)	0.014 (SE = 0.047)	−0.078, 0.106	*t*(714) = 0.295, *p* = 0.768
	Wind direction (cosine)	0.100 (SE = 0.365)	−0.617, 0.817	*t*(714) = 0.273, *p* = 0.785
	Wind direction (sine)	0.286 (SE = 0.376)	−0.452, 1.024	*t*(714) = 0.760, *p* = 0.448
Unspecified headache (AAN)				
	Age	0.019 (SE = 0.028)	−0.036, 0.074	*t*(712) = 0.680, *p* = 0.497
	Gender (Female)	−0.498 (SE = 0.022)	−0.541, −0.456	*t*(712) = −23.000, ***p* < 0.001**
	Gender (Male)	−0.697 (SE = 0.070)	−0.834, −0.560	*t*(712) = −9.986, ***p* < 0.001**
	Average temperature (degrees Celsius)	0.006 (SE = 0.068)	−0.127, 0.138	*t*(712) = 0.082, *p* = 0.935
	Diurnal temperature range (degrees Celsius)	0.010 (SE = 0.183)	−0.349, 0.369	*t*(712) = 0.054, *p* = 0.957
	Day-to-day temperature change (degrees Celsius)	−0.171 (SE = 0.512)	−1.176, 0.834	*t*(712) = −0.333, *p* = 0.739
	Average rainfall (L/m^2^)	0.042 (SE = 0.149)	−0.251, 0.335	*t*(712) = 0.279, *p* = 0.780
	Average wind speed (m/s)	0.002 (SE = 0.667)	−1.308, 1.311	*t*(712) = 0.003, *p* = 0.998
	Wind gusts (m/s)	0.009 (SE = 0.323)	−0.624, 0.643	*t*(712) = 0.029, *p* = 0.977
	Sunshine hours	0.031 (SE = 0.257)	−0.473, 0.535	*t*(712) = 0.120, *p* = 0.904
	Daily barometric pressure range (hPa)	−0.023 (SE = 0.299)	−0.611, 0.564	*t*(712) = −0.077, *p* = 0.938
	Day-to-day barometric pressure change (hPa)	0.148 (SE = 0.635)	−1.099, 1.396	*t*(712) = 0.233, *p* = 0.816
	Wind direction (cosine)	2.982 (SE = 5.436)	−7.690, 13.653	*t*(712) = 0.549, *p* = 0.583
	Wind direction (sine)	2.588 (SE = 5.235)	−7.691, 12.866	*t*(712) = 0.494, *p* = 0.621

*Models notation in parenthesis: ETSX (error, trend, seasonality). ARIMAX: AutoRegressive Integrated Moving Average with external regressors; AM: AutoRegressive order; I: Integrated order; MA: Moving Average order; S: Seasonal order component; m: Seasonal period; LAG order; SE: Standard Error; 95% CI: 95% confidence interval; ANN: Additive error, No trend, No seasonality; AAN: Additive error, Additive trend, No seasonality. ^a^significant if p < 0.05 (shown in bold).*

Across the 4-year forecasting horizon, the models projected a moderate increase in the number of headache consultations, primarily driven by unspecified headache subtypes, whereas cluster headache and tension-type headache consultations were projected to remain stable (Fig. 2).

**Fig. 2.** Time-series forecast. Forecasted trends for 2024–2027 were generated using ETSX and ARIMAX models. A moderate increase was projected in the total number of unspecified headache consultations, whereas cluster- and tension-type headaches showed stable patterns. The shaded areas represent 95% confidence intervals of the model predictions.

## 4. Discussion

### 4.1 Principal findings

This 14-year time-series analysis, conducted to examine whether climatic variables are associated with weekly primary care consultations for cluster headache, tension-type headaches, and unspecified headaches, revealed that only a small subset of climatic variables showed independent associations with headache consultations. Moreover, the nature of these associations differed across the headache subtypes. The ARIMAX model best captured the dynamics of tension-type headache (TTH), whereas the ETSX model provided superior predictive accuracy for overall headache, unspecified headache, and cluster headache (CH), in agreement with the model comparison metrics from the supplementary analyses.

Among all headache types, sex was the most consistent predictor. In the TTH series, female sex was the only demographic factor significantly associated with an increased number of consultations, whereas male sex was not significant. Average temperature showed a modest but significant protective effect, reducing the weekly number of TTH consultations.

For CH, the male sex was significantly associated with reduced consultation frequency, whereas no climatic variable reached significance in the final ETSX model. In the overall and unspecified headache models, sex remained a significant regressor; however, the negative coefficients observed for both the female and male sex should be interpreted in relation to the model intercept and do not indicate a higher consultation burden in men. These findings are consistent with the descriptive data showing a predominance of female consultations. However, no meteorological variable showed an independent association. Overall, these results indicate that the influence of weather on headache consultations is weaker and more subtype-specific than traditionally assumed and is largely overshadowed by demographic factors, particularly sex.

### 4.2 Comparison with previous studies

The present findings refine and partially challenge previous evidence suggesting the strong influence of meteorological variables on headache occurrence [10, 11, 12, 13, 14, 15, 17]. Earlier studies have associated temperature variability, humidity, and barometric pressure changes with increased risk of migraine, TTH, or CH attacks [10, 11, 12, 13, 14, 15, 17]. However, many of these investigations relied on patient-reported triggers, smartphone-based symptom diaries, or short observational windows [19, 20], which may amplify the perceived relevance of weather-related factors.

Our results diverge from population studies indicating a strong climatic modulation of CH periods, particularly through temperature variation [13, 17], because temperature, wind, rainfall, pressure, and sunshine hours were not significant predictors of CH in our long-term primary care dataset. Similarly, previous work linking barometric pressure changes to TTH exacerbations [11, 32] was not corroborated by our models, in which pressure-related variables were consistently nonsignificant.

Sex-related findings in our models echo long-established epidemiological patterns: female sex was associated with higher TTH consultation rates, whereas male sex was not independently associated, in line with global burden estimates [8]. Conversely, the protective effect of male sex on CH aligns with contemporary CH epidemiology [25].

The disparity observed in this study between the number of men and women seeking consultations for headaches is likely partly related to sex differences in healthcare-seeking behaviors. Women have consistently been shown to engage more frequently in healthcare services when experiencing pain, particularly in primary care settings. Psychosocial factors, including heightened concern about symptoms, gender-related expectations, and emotional distress, may further increase the likelihood of women seeking medical attention for headache-related complaints. Recent population-based evidence indicates that women are more likely than men to seek healthcare across a wide range of symptoms and conditions, even after accounting for sociodemographic factors and health status. This supports the interpretation that consultation-based data may reflect behavioral patterns in healthcare utilization rather than true differences in disease occurrence alone [33].

Overall, the present study suggests that climatic factors exert a weaker influence on healthcare utilization for headaches than previously hypothesized, particularly when analyzed over long temporal frames using objective registry data [18, 19, 20, 22].

### 4.3 Mechanistic considerations

Although most climatic variables did not independently predict headache consultations, the few significant associations—temperature and wind direction for TTH—may reflect underlying neurophysiological pathways described in biometeorological research. Variations in ambient temperature can influence autonomic balance, vascular tone, and the thermoregulatory mechanisms involved in trigeminovascular activation [7, 10, 16]. Directional changes in wind flow are often accompanied by transitions in barometric pressure, humidity, and ionization patterns, which may modulate pericranial muscle tension or trigeminal afferent excitability [18, 22, 34].

The lack of climatic associations in CH contrasts with hypotheses involving hypothalamic sensitivity to environmental cues and circannual rhythmicity [21]. It is possible that individual-level triggers, such as sleep irregularities, stress, or behavioral changes, exert a much stronger influence on CH attacks than population-level meteorological patterns.

The post-2020 increase in overall headache consultations observed in the present study likely reflects pandemic-related psychosocial factors rather than meteorological effects, including increased stress, altered sleep patterns, and emotional distress, all of which are known contributors to headache frequency [35, 36]. In addition, increased healthcare utilization following the COVID-19 pandemic has been reported not only for headaches but also for other orofacial pain conditions [37, 38].

### 4.4 Clinical and public health implications

From a clinical standpoint, these findings provide a nuanced perspective on the role of weather in headache management. Although biometeorological influences exist, their magnitude appears modest and may not justify strict behavioral restrictions for most patients. Furthermore, from a clinical perspective, it is important to recognize that patients often attribute their headaches to weather-related factors; however, as shown in this study, climatic variables appear to play a more limited role than previously assumed, and patient education should therefore aim to help deconstruct such beliefs and expectations [23, 32]. Nevertheless, clinicians should incorporate climatic factors as secondary modulators, particularly in individuals who report personal meteorosensitivity, as suggested in the literature [23, 32].

The validated ETSX and ARIMAX models offer a foundation for developing forecasting tools capable of anticipating fluctuations in consultation demand, particularly given the strong demographic patterns observed. Such predictive models may help optimize primary care workload distribution, support patient education, and guide individualized self-management strategies during periods of abrupt weather changes.

However, the present results caution against overstating the epidemiological impact of weather factors on headache. Personalized profiling, as advocated in recent biometeorological frameworks [22], may better capture the clinically relevant subsets of patients sensitive to climatic variations.

In addition, adverse weather conditions may influence healthcare-seeking behavior, potentially delaying or discouraging primary care visits, independent of headache severity. As individual-level data on access patterns or delayed consultations were not available, this potential behavioral confounding factor could not be directly assessed and should be considered when interpreting consultation-based outcomes.

### 4.5 Limitations and future directions

Several limitations of this study must be acknowledged. First, reliance on primary care diagnostic codes may underrepresent true headache prevalence and may not fully capture clinical severity or attack characteristics. Second, meteorological data were obtained from a single AEMET station, limiting the assessment of microclimatic variability within the catchment area. Third, the study did not include potentially interacting environmental exposures, such as air pollution, allergens, or noise, some of which have been linked to increases in headache-related emergency visits [39, 40]. Fourth, the absence of patient-reported outcomes prevented us from examining subjective meteorosensitivity, attack timing, and individual trigger perceptions. Another limitation regarding climatic variables was the absence of humidity data, despite evidence in the literature suggesting that humidity may be associated with headache occurrence [3, 6, 10, 11, 12, 13, 14, 17, 18, 19, 20, 25]. An important methodological limitation of this study is the reliance on primary care consultation data as a proxy for headache occurrence. Not all headache episodes lead to a medical visit, particularly among individuals who self-manage their symptoms, experience recurrent or habitual headaches, or perceive limited benefit from repeated consultations. As a result, consultation counts may not fully reflect the true temporal distribution or frequency of headache episodes in the underlying population. This limitation is particularly relevant when interpreting associations with climatic variables, as the observed time-series patterns primarily represent healthcare-seeking behavior rather than the actual incidence of headache attacks.

Future studies should integrate multi-station meteorological data, incorporate pollutant measures, and combine registry-based models with smartphone-based symptom tracking. Machine learning approaches and individualized trigger profiling may substantially improve the predictive accuracy and clarify the mechanisms linking climate and headache exacerbations. In clinical practice, it is common for patients to report worsening of symptoms associated with cold or wind flow. It would be interesting to correlate these data with more specific diagnoses, such as cervicogenic headaches or headaches secondary to temporomandibular disorders.

## 5. Conclusions

This 14-year primary care study demonstrated that demographic factors, particularly sex, were more strongly associated with headache consultation patterns than climatic variables. Among the meteorological factors, only average temperature and wind direction showed small independent associations with tension-type headache consultations, whereas no climatic variables were significantly associated with cluster headache or unspecified headache consultations. These findings suggest that the epidemiological impact of climate on headache patterns may be more limited and subtype-specific than previously assumed.

The validated ETSX and ARIMAX models highlight the feasibility of using time-series forecasting to monitor consultation trends and support healthcare planning. Integrating biometeorological monitoring with demographic and behavioral data may enhance the predictive accuracy and facilitate the personalized management of patients who exhibit weather-related symptom fluctuations.

## Supplementary Material

Supplementary material associated with this article can be found, in the online version, at https://files.jofph.com/files/article/2066697965739622400/attachment/Supplementary%20material.docx.


