# Supplementary material for: The impact of climatic factors on headache patterns: a 14-year time series analysis of cluster and tension-type headaches in primary care
Source: J Oral Facial Pain Headache. 2026 Jul 12;40(4):63–75. doi: 10.22514/jofph.2026.045 (PMC13434138; doi:10.22514/jofph.2026.045)
Supplement: Supplementary file 1 [file Supplementary-material.docx]

Supplementary material

Supplementary Table 1. Time series model selection.

|  | | Model parameters | RMSE | SMAPE | MASE |
| --- | --- | --- | --- | --- | --- |
| Overall | | | | | |
|  | ETSX model | M, N, N | 22.845 | 32.002 | 1.987 |
|  | ARIMAX model | AR0, I0, MA2, SAR0, SI0, SMA0, LAG1, m52 | 10.300 | 62.040 | 2.008 |
| Cluster headache | | | | | |
|  | ETSX model | M, N, N | 0.743 | 166.739 | 1.828 |
|  | ARIMAX model | AR0, I0, MA1, SAR0, SI0, SMA0, LAG1, m52 | 0.603 | 197.733 | 1.171 |
| Headache | | | | | |
|  | ETSX model | M, M, N | 31.720 | 43.735 | 3.912 |
|  | ARIMAX model | AR0, I0, MA1, SAR0, SI0, SMA0, LAG1, m52 | 10.314 | 72.271 | 2.589 |
| Tension-type headache | | | | | |
|  | ETSX model | M, N, N | 3.014 | 142.917 | 0.871 |
|  | ARIMAX model | AR0, I0, MA3, SAR0, SI0, SMA0, LAG1, m52 | 1.668 | 125.980 | 0.692 |

Models notation: ETSX (error, trend, seasonality), letters M: Multiplicative and N: Non-present. ARIMAX: AutoRegressive Integrated Moving Average with external regressors; AM: AutoRegressive order; I: Integrated order; MA: Moving Average order; S: Seasonal order component; m: Seasonal period; LAG order; RMSE: Root mean squared error; SMAPE: Symmetric Mean absolute percentage error; MASE: Mean absolute scaled error.

Supplementary Table 2. ETSX models selection.

|  | Model parameters | RMSE | SMAPE | MASE |
| --- | --- | --- | --- | --- |
| Overall | | | | |
|  | M, N, N (naive model) | 22.845 | 32.002 | 1.987 |
|  | ANN | 4.321 | 27.952 | 0.873 |
|  | AAN | 3.922 | 25.659 | 0.797 |
| Cluster headache | | | | |
|  | M, N, N (naive model) | 0.743 | 166.739 | 1.828 |
|  | ANN | 0.625 | 196.884 | 1.377 |
|  | AAN | 0.626 | 197.116 | 1.314 |
| Headache | | | | |
|  | M, M, N (naive model) | 31.548 | 43.633 | 3.896 |
|  | ANN | 5.492 | 35.289 | 1.359 |
|  | AAN | 4.103 | 29.890 | 1.047 |

Models notation letters A: Additive, M: Multiplicative and N: Non-present; RMSE: Root mean squared error; SMAPE: Symmetric Mean absolute percentage error; MASE: Mean absolute scaled error; ANN: Additive error, No trend, No seasonality; AAN: Additive error, Additive trend, No seasonality.

Supplementary Table 3. ARIMAX and ETSX models evaluation.

|  | Kolmogorov-Smirnov test (*p* value) | Ljung-Box test (*p* value) |
| --- | --- | --- |
| Overall | **0.001**^a^ | 0.845 |
| Cluster headache | **<0.001**^a^ | 0.068 |
| Headache | **0.002**^a^ | 0.598 |
| Tension headache | **<0.001**^a^ | 0.649 |

^a^significant if *p* < 0.05 (shown in bold).
